# Pathological Osteoclasts and Precursor Macrophages in Inflammatory Arthritis

**DOI:** 10.3389/fimmu.2022.867368

**Published:** 2022-04-08

**Authors:** Tetsuo Hasegawa, Masaru Ishii

**Affiliations:** ^1^ Department of Immunology and Cell Biology, Graduate School of Medicine and Frontier Biosciences, Osaka University, Osaka, Japan; ^2^ Division of Rheumatology, Department of Internal Medicine, Keio University School of Medicine, Tokyo, Japan; ^3^ World Premier International Research Center Initiative (WPI)-Immunology Frontier Research Center, Osaka University, Osaka, Japan; ^4^ Laboratory of Bioimaging and Drug Discovery, National Institutes of Biomedical Innovation, Health and Nutrition, Osaka, Japan

**Keywords:** macrophage, osteoclast, rheumatoid arthritis, single-cell RNA sequencing, intravital imaging

## Abstract

Macrophages comprise a variety of subsets with diverse biological functions, including inflammation, tissue repair, regeneration, and fibrosis. In the bone marrow, macrophages differentiate into multinucleated osteoclasts, which have a unique bone-destroying capacity and play key roles in physiological bone remodelling. In contrast, osteoclasts are also involved in inflammatory bone erosion in arthritis and it has been unclear whether the osteoclasts in different tissue settings arise from similar monocytoid precursors and share similar phenotypes. Rapid progresses in the sequencing technologies have provided many important insights regarding the heterogeneity of different types of osteoclasts. The application of single-cell RNA sequencing (scRNA-seq) to the osteoclast precursor-containing macrophages enabled to identify the specific subpopulation differentiating into pathological mature osteoclasts in joints. Furthermore, an intravital imaging technology using two-photon microscopy has succeeded in visualizing the real-time dynamics of immune cells in the synovial microenvironment. These technologies together contributed to characterize the unique macrophages in the inflamed synovium, termed “arthritis-associated osteoclastogenic macrophages (AtoMs)”, causing the pathological bone destruction in inflammatory arthritis. Here, we review and discuss how novel technologies help to better understand the role of macrophages in inflammatory arthritis, especially focusing of osteoclastogenesis at the pannus-bone interface.

## Introduction

Macrophages are distributed throughout the body and possess a variety of biological activities, contributing to tissue homeostasis and a broad spectrum of pathogenesis in autoimmune/autoinflammatory diseases. One of the most unique features of macrophages is their potential to fuse with each other to differentiate into multinucleated osteoclasts in the bone marrow (BM) cavity. Under physiological conditions, they support steady-state bone remodeling together with bone-forming cells, osteoblasts. On the other hand, osteoclasts are also involved in pathological joint destruction at the synovium-bone interface, called “bare area”, in patients with rheumatoid arthritis (RA). Although extensive studies of osteoclast precursor (OP)-containing population in the BM and osteoclast-like cells derived from BM macrophages have been done (1, 2), the microenvironment of inflamed synovium is highly different from the BM in terms of surrounding cell populations, cytokine milieu, and tissue structures. Therefore, a number of fundamental questions regarding pathological bone destruction remained unanswered, such as the phenotype of *in situ* OP populations in the synovium and the origin of pathological osteoclasts in arthritis.

Recent advances in dissecting the inflamed synovium from arthritic mice gave us great insights into macrophages and OPs in the pannus of synovial tissues, the actual site of bone destruction in arthritis (3). In addition, scRNA-seq analysis has succeeded in identifying small subsets in heterogeneous immune cell populations within the joint tissue. Furthermore, the development of intravital imaging system using two-photon microscopy enabled us to to directly analyze the dynamics of osteoclasts and immune cells *in vivo* (4–7). These three skills in combination contributed immensely to better understand of the pathogenesis of arthritic bone erosion and this review introduces recent advances in understanding the role of monocytes/macrophages in inflammatory arthritis.

## Differentiation Trajectory of Pathological Osteoclasts in Joints

**Figure 1:**
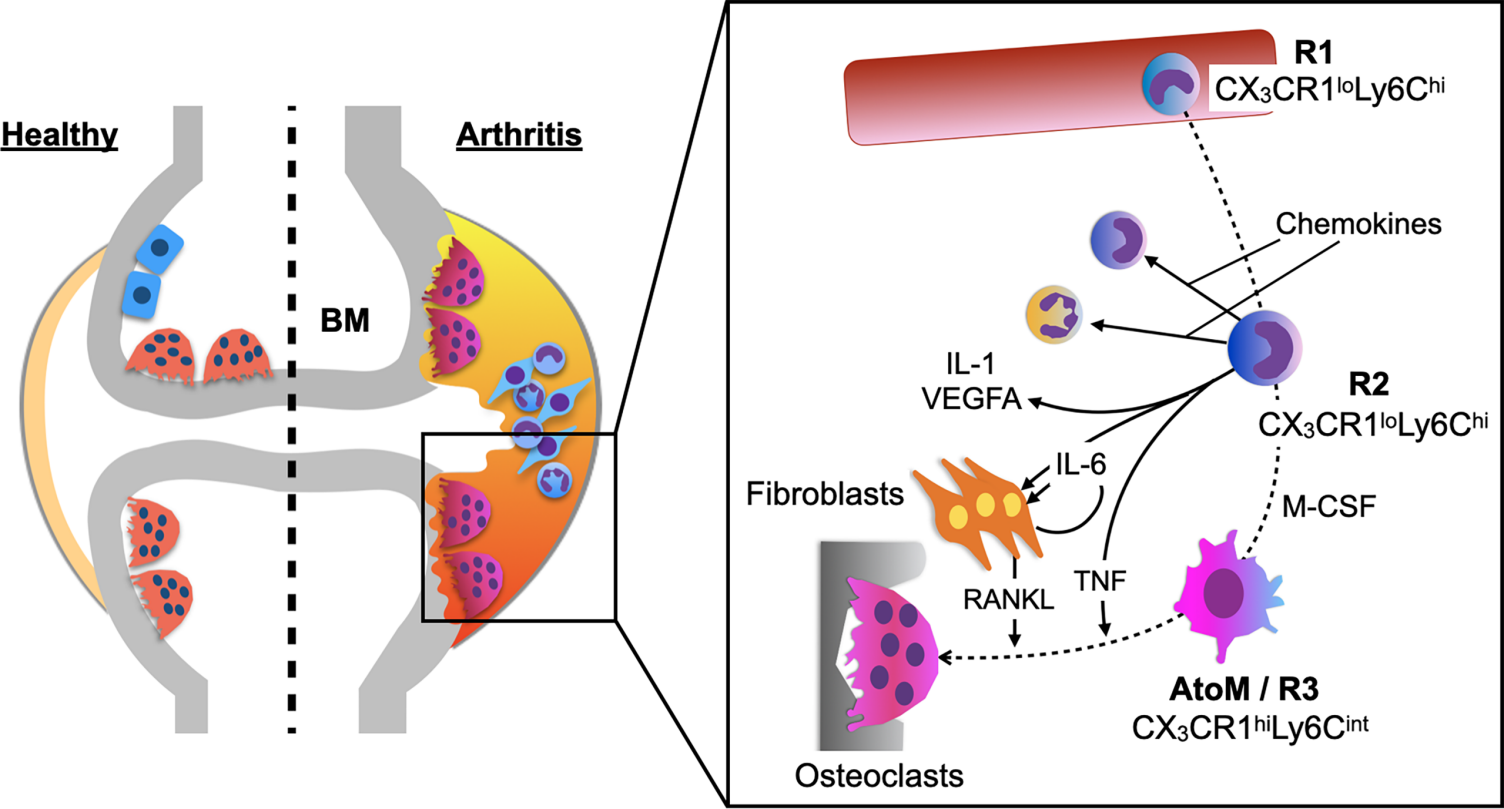
Differentiation trajectory of pathological osteoclasts in arthritis. BM-derived CX_3_CR1^lo^Ly6C^hi^ cells (R1) ingress into the synovium (R2) and produce inflammatory cytokines, chemokines, and VEGFA. Abundant M-CSF in the inflamed synovium induces maturation of some R2 cells into osteoclast precursors, arthritis-associated osteoclastogenic macrophages (AtoMs/R3 cells). When AtoMs localize adjacent to the bone surface, simultaneous stimulation with RANKL and TNF promotes osteoclast formation, leading to joint destruction in arthritis.

A healthy synovial membrane consists of scattered macrophage-like cells within a fibroblast stromal tissue and is relatively acellular. However, in RA, the synovial membrane becomes hypertrophic with a variety of immune cells and bone erosion is the central hallmark of the disease, which takes place predominantly at the pannus-bone interface called “bare area”. The precise protocol to purely isolate the pannus tissue from the inflamed synovium in an arthritic mouse model has been documented in the previous study (3). *Ex vivo* culture system of the pannus tissue revealed that OPs are within the CX_3_CR1^+^ cells and CX_3_CR1^hi^Ly6C^int^F4/80^+^I-A/I-E^+^ macrophages had the highest capacity to differentiate into osteoclasts among all the monocytoid cells in the pannus tissue. To elucidate the origin of this specific cell subset, which was designated as arthritis-associated osteoclastogenic macrophages (AtoM) (3), BM chimeric models and a parabiosis model of CX_3_CR1-EGFP/TRAP-tdTomato double transgenic mice were used. The results showed that AtoMs and pathological osteoclasts are derived from blood monocytes and not from synovium-resident macrophages (**Figure 1**).

A detailed analysis of the differentiation trajectory of osteoclasts under arthritic conditions showed that CX_3_CR1^lo^Ly6C^hi^ cells in the blood (R1) ingress into the synovium (R2) and differentiate into CX_3_CR1^hi^Ly6C^int^ cells (R3). Global transcriptomic analysis showed that R2 cells predominantly expressed transcripts encoding chemokines, inflammatory cytokines (*Il1*, *Il6*, and *Tnf*), and *Vegfa*, while R3 cells highly expressed osteoclast marker genes, such as *Mmp9*, *Acp5*, *Ctsk*, *Atp6v0d2*, and *Ppargc1b*. Together, the study showed that monocytes acquire a highly inflammatory phenotype when they ingress into the synovium, and abundant M-CSF in the synovial microenvironment induces maturation of some of these cells into AtoMs, leading to osteoclast formation at the pannus-bone interface (**Figure 1**) (3).

## Cytokines Involved in Pathological Osteoclast Formation

Receptor activator of nuclear factor kappa-B ligand (RANKL) plays a critical role in the articular bone destruction in arthritic mouse models (8), and in RA (9–11). Nevertheless, the primary source of RANKL in the synovial microenvironment has been controversial. A study analyzing conditional knockout mice of RANKL in fibroblasts (Col6a1-Cre) and T cells (Lck-Cre) showed that RANKL expression in fibroblasts contributes mainly to the bone erosion in arthritis (12). Moreover, the analysis of each cell types in the inflamed synovium of arthritic mice showed that RANKL expression in fibroblasts is almost 400 times higher than those in leukocytes (3). Together, these results indicate that fibroblasts are the major source of RANKL in the synovial microenvironment (**Figure 1**).

Various inflammatory cytokines are known to contribute to the bone erosion in arthritis, including IL-1, IL-6, and tumor necrosis factor (TNF). TNF is mainly expressed on macrophages and T cells in the inflamed synovium, which activates TNF receptors type 1 and 2. TNF contributes to osteoclast formation by inducing paired Ig-like receptor-A, a costimulatory receptor for RANK, on OPs (13). Since TNF did not induce osteoclastogenesis when it was administered *in vivo* to RANK knockout mice (14), the permissive levels of RANKL is indispensable for TNF to function against OPs. RANKL priming several days prior to TNF stimulation induces maximal osteoclast formation effect in BM macrophages, while TNF does not promote osteoclast formation when added simultaneously with RANKL (13, 15). In contrast, simultaneous stimulation of RANKL and TNF significantly promotes osteoclastogenesis of OPs in the inflamed synovium, AtoMs (3), indicating that AtoM is distinct from conventional OP populations in the BM and TNF has dual functions in triggering inflammatory osteolysis by inducing inflammation and directly affecting OPs to differentiate into mature osteoclasts in the synovium.

Although IL-1β alone does not directly induce osteoclast formation (16), it can induce osteoclastogenesis from TNF pre-activated OPs by the process independent of NF-κB p50 and p52 (17). In addition, IL-1β enhances stromal cell expression of RANKL, playing an indirect role in osteoclast formation (18). IL-6/sIL-6R directly induce RANKL expression in fibroblasts in RA and this is mediated by the Janus kinase/STAT signalling pathway (19).

## Single Cell RNA-Seq Analysis of Osteoclast Precursor Macrophages in the Joint Tissue

Single cell RNA-seq (scRNA-seq) analysis has been applied to synovial macrophages both in mice (20) and human patients (21), revealing heterogeneous subsets that participate in joint inflammation. When this technique was applied specifically to the OP population within the inflamed synovium, the precise number and small subpopulation of macrophages differentiating into pathological osteoclasts *in situ* was estimated. ScRNA-seq analysis of the synovial OP population, AtoMs, showed that about 10% of AtoMs (approximately 1,000 cells per mouse) differentiate into mature osteoclasts in the inflamed synovium (3). A statistical method called pseudotime trajectory analysis, which shows developmental processes from a cell population at asynchronous stages, revealed that AtoMs consist of cells that are under continuous developmental processes into mature osteoclasts, while a minority of cells differentiate into another cell type, which expresses *Nrp1, CD36*, and *C5ar1* (7). The small subpopulation of OPs within AtoMs specifically expressed *FoxM1*, which is a multifaceted transcription factor that plays a prominent role in carcinogenesis by rendering tumour cells to be more aggressive and invasive, leading to metastasis (22). There is a correlation between tumour and pannus tissues that both invade the surrounding tissue and can erode the bone surface. FoxM1 inhibitor and depletion of *Foxm1* in arthritic mice suppressed the articular bone erosion *in vivo*, suggesting that FoxM1 constitutes a potential target for RA treatment. The master regulator of osteoclastogenesis, *Nfatc1*, was also highly expressed in the small subpopulation of OPs in AtoMs, indicating its role in pathological bone destruction. However, expression levels of *Nfatc1* were comparable in bulk RNA-seq analysis between AtoMs and R2 cells, and further investigation is required to analyse the functional contribution of *Nfatc1* in pathological osteoclastogenesis of AtoMs (3).

## Intravital Imaging System for the Synovial Tissue

**Figure 2:**
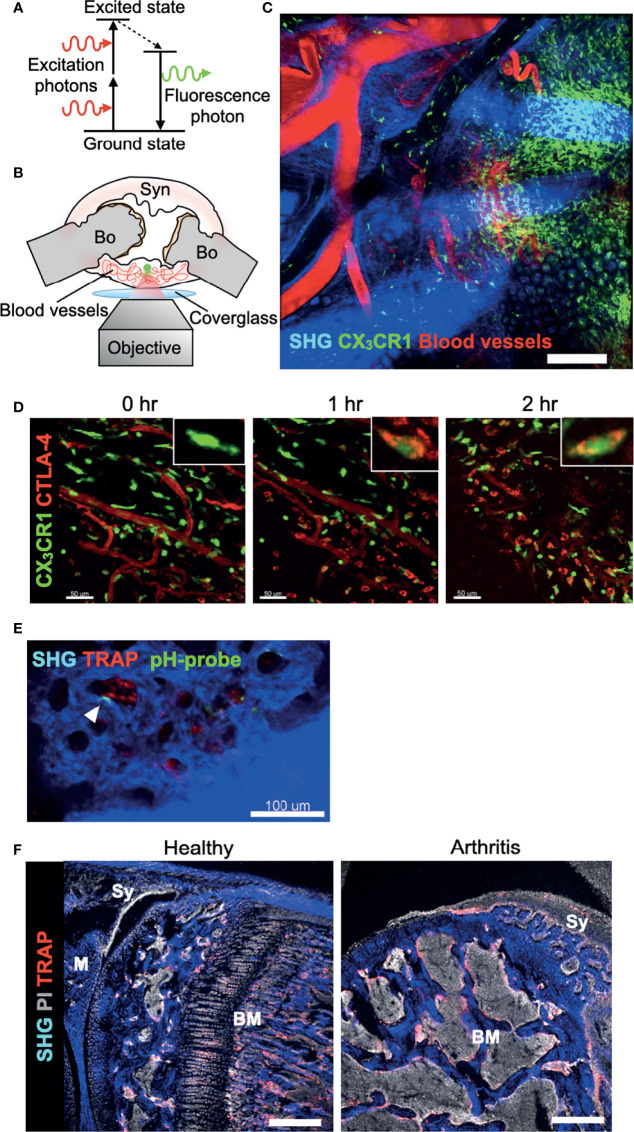
Real-time imaging of the synovium *in vivo* using multi-photon microscopy. **(A)** Basic mechanism of two-photon excitation. Two-photon excitation occurs when a fluorophore absorbs two photons simultaneously. Each photon possesses about half of the energy required to excite the fluorophore. **(B)** Schematic of preparation of exposed synovium, with a coverglass and microscope objective positioning. The green dot shows the area of two-photon excitation. Bo, Bone; Syn, synovium. **(C)** Tile scan image of the inflamed synovium of CX_3_CR1-EGFP transgenic mice taken by two-photon microscopy. Blood vessels are visualized *via* intravenous injection of CTLA-4 Ig labeled with AF647 (red). Collagen fibers are visualized by second harmonic fluorescence generated from two-photon excitation (blue). Scale bar: 200 μm. **(D)** Time-lapse imaging of the inflamed synovium of CX_3_CR1-EGFP transgenic mice. Intravenously injected CTLA-4 Ig (red) extravasates and binds to CX_3_CR1^+^ macrophages (green) after one hour. Scale bars: 50 μm. The MIPs of two-dimensional image stacks of vertical synovial slices are shown. **(E)** Intravital images of the third meta phalangeal joint of the CIA TRAP-tdTomato transgenic mice (red) after pHocas-3 (green) injection. Bar, 100 μm. **(F)** Propidium iodide (PI) and TRAP staining fluorescence were visualized by single-photon excitation, while second harmonic generation was produced by two-photon excitation to visualize the bone tissue of the knee joint section. Scale bar: 300 μm. BM, bone marrow; M, meniscus; Sy, synovium.

Although conventional methods, such as flow cytometry, micro-computed tomography, and histomorphological analyses, can give us information on the bone structures and molecular expression patterns, intravital information on dynamic cell movements and cellular interactions is not available. An intravital imaging system using two-photon microscopy provided valuable insights into the intravital behaviour and function of immune cells in a variety of organs (23–26). Two-photon microscopy uses two near-infrared photons for exciting the fluorescent molecule to observe the cellular dynamics of deep tissues (100–1,000 μm), to minimize photobleaching and phototoxicity, and to use a nonlinear optical process called second-harmonic generation (SHG) to visualize collagen fibers (**Figure 2A**). The advantages and disadvantages of different modalities used for bone and joint researches are listed in **Table 1**.

**Table 1:** Comparison of different modalities used for bone and joint researches.

Method	Advantages	Disadvantages
Confocal microscopy (CM)	➢Ideal for moderate tissue penetration with simultaneous, multicolor imaging ➢High spatial and temporal resolutions ➢Faster acquisition times compared to MPM	➢Photobleaching and phototoxicity ➢Limitation in thickness of tissues because of light scattering
Multi-photon microscopy (MPM)	➢Ideal for deep tissue penetration ➢Efficient light detection ➢Excitation occurs only at the focal plane, reducing phototoxicity and photobleaching ➢Detection of bone and fibrous tissue by second harmonic generation (SHG) ➢Intravital cellular dynamics and interactions can be observed	➢Higher costs in microscopy purchase/maintenance ➢Artifacts caused by autofluorescence ➢Longer acquisition times compared to CM ➢Limitation in imaging more than four colors due to lack of laser availability ➢Requires skills in handling living animals
MicroCT	➢Three-dimensional visualization of whole bone architecture ➢Quantitative measurements of tissue density ➢Rapid compared to thin sectioning	➢No information on the cellular level ➢No information on the molecular level
Histochemistry	➢Inexpensive ➢Highly specific for individual molecules ➢Many different markers can be combined ➢Can be used for light, confocal, or electronic microscopy	➢The quality of immunolabelling depends on the specificity of the antibody ➢Time and labor consuming ➢Limited to one plane of the section

While the intravital imaging of macrophages (27) and cancer cells (28) in the BM cavity has been well performed, visualization of deep areas of synovial tissue has been difficult for several reasons. First, the hypertrophied synovial tissue is composed of multiple layers with different refraction indexes, including lining and sublining layers, which limits the depth of observation. Second, the visual field of peripheral joints can easily drift in accordance with respiratory movement. Therefore, we decided to expose small joints of the arthritic mice, including wrists and metacarpophalangeal joints, to overcome the first obstacle. Then, we fixed the region of interest to a cover glass and observed the area with inverted microscopy to overcome the second obstacle (**Figure 2B**). A wide field of view can be obtained by tile scan imaging, and monocytes/macrophages are directly visualized *in vivo* in the synovium of a CIA mouse model (**Figure 2C**) (29). When a biological agent, cytotoxic T-lymphocyte-associated protein 4 (CTLA-4) Ig, is fluorescently labelled, we could track the intravital distribution of CTLA-4 Ig under arthritic conditions and observe the binding capacity of CTLA-4 Ig to CX_3_CR1^+^ macrophages in the inflamed synovium (**Figure 2D**). This state-of-the-art technique allows us to observe how the binding of an agent affects the behaviour of macrophages *in situ*.

When the depth of the observation area reaches around 50–100 μm from the synovial surface, mature osteoclasts that resorb the bone matrix can be observed at the pannus–bone interface (29). Osteoclasts are fluorescently labelled in TRAP-tdTomato mice (5) and bone tissue was visualized by SHG. CX_3_CR1-EGFP^+^ cells as well as CX_3_CR1-EGFP/TRAP-tdTomato double positive cells were detected at the pannus-bone interface and pathological osteoclasts were making 50-μm-diameter resorption pits (29) (**Figure 2E**). Furthermore, the acidic region caused by functional osteoclasts could be detected by using a pH-sensing chemical fluorescent probe (30). A bisphosphonate group of this probe attaches to the bone surface and boron-dipyrromethene dye emits fluorescence, which has high environmental stability *in vivo*. Combining these techniques, intravital imaging showed that mature osteoclasts were actively resorbing the articular bone without migrating on the bone surface (**Figure 2E**) (29). These results contrast with the osteoclasts under homeostatic conditions in the BM cavity, which are in close contact with osteoblasts and migrate on the bone surface (31). Together, this intravital imaging protocol for the synovium can serve as a platform for exploring the dynamics of immune cells and osteoclasts in the synovial microenvironment. Further studies are required to elucidate which cell subsets and cytokine milieu in the synovium and BM are responsible for the distinctive bone-resorbing behaviour of these osteoclasts.

Another application of two-photon microscopy in the research of bone and joint diseases is the usage of SHG in immunohistochemistry. By combining the images taken by single- and two-photon lasers, we can simultaneously obtain a wide variety of fluorescence signals in combination with SHG signals that detects bone and collagenous synovial layer (**Figure 2F**) (3).

## Clinical Implications

Identification of osteoclastogenic macrophages in the joint, AtoMs, gives us several clues for the new treatment strategy for inflammatory osteolytic diseases. Since AtoMs originate from blood monocytes, chemokines involved in their ingress into the synovial tissue are potential targets, such as CCR2 and CX_3_CL1 (fractalkine). M-CSF and FoxM1 are also shown to be essential for OP formation both *in vitro* and *in vivo*, thereby constituting potential targets for bone destruction in arthritis. Simultaneous stimulation with RANKL and TNF most efficiently induced osteoclastogenesis of AtoMs, implying that TNF-inhibitors possess a direct effect in inhibiting pathological osteoclast formation. In accordance with this finding, TNF blockade can prevent progressive joint damage in patients with RA who have a clinical response as well as in those who do (32, 33), and combination therapy of anti-RANKL monoclonal antibody and biological agents showed the efficacy on radiographic progression in RA (10). Although there’s no study that specifically analysed the effect of combination therapy of anti-RANKL monoclonal antibody and TNF-inhibitor, this may be one of the options for patients with progressive bone destruction despite of the use of single biological agent. Although IL-6 didn’t have a direct effect on osteoclastogenesis of AtoMs, IL-6/sIL-6R induce RANKL expression in fibroblasts in RA (19). Therefore, IL-6 may function indirectly in osteoclastogenesis by promoting secretion of cytokines essential for osteoclast formation from stromal cells. Further investigation of IL-6 involvement in osteoclastogenesis with human synovial samples is indispensable to make a final conclusion on this issue.

An important question still remains elusive why synovitis in some diseases, such as RA, leads to bone destruction and others do not. Is it just a matter of the duration and intensity of synovitis? Or are they totally different in terms of their pathogenesis? Although we do not have any clear answer to this question, the differences in the cytokine milieu of synovial microenvironment in each disorder may give us a clue. In diseases that lead to bone destruction, such as RA and septic arthritis, TNF is abundantly expressed in the synovium and causes not only inflammation but also osteoclastogenesis of AtoMs. In addition, M-CSF is present in great quantity to support synovial OP formation at the pannus-bone interface. On the other hand, TNF is not involved in the pathogenesis of SLE, as demonstrated by the possible negative effect of TNF-inhibitors in this disorder, and SLE seldom causes devastating bone erosion. Localization of the inflammation can also play a key role in inflammatory osteolysis. Although TNF is highly involved in the pathogenesis of psoriatic arthritis, enthesis is the representative location of inflammation and it lacks enough capillary networks and the cytokine milieu required for monocytic cells to differentiate into OPs and mature osteoclasts.

AtoMs are distinctive from physiological OPs in the BM in that they express cell surface markers for antigen presentation, such as CD80/86 and MHC class II, and CD11c. Since several studies showed that immature dendritic cells can differentiate into osteoclasts (34,35), these synovial macrophages may be playing a role in antigen presentation in the tertiary lymphoid tissue in the synovium. In fact, fluorescently labelled CTLA4-Ig binds to AtoMs after systemic injection in arthritic mice (29), implying that CTLA4-Ig targets these macrophages in the tertiary lymphoid structure to inhibit auto-antigen presentation in the inflamed synovium. Antigen presenting capacity as well as the expression of CD83, an activation marker for antigen presenting cells, of AtoMs should be assessed in the future.

## Conclusion

Advances in synovial dissecting procedures, sequencing technologies, and intravital imaging system using multi-photon microscopy have contributed immensely to elucidate the phenotype of macrophages specifically involved in the arthritic bone destruction. Compared to other tissues involved in autoinflammatory/autoimmune diseases, the most characteristic feature of arthritis is bone erosion caused by the final effector cells, osteoclasts. Single cell RNA-seq analysis of the OP-containing macrophages in the joint tissue succeeded in specifying the small subpopulation differentiating into mature osteoclasts at the pannus-bone interface and intravital imaging technique revealed their real-time dynamics. While personalized medicine for autoimmune diseases, including RA, remains a goal out of our reach, novel findings with regard to the differentiation trajectory and distinct phenotype of osteoclastogenic macrophages in the synovium will facilitate us to achieve optimization on treatment for individual patient, and support us to achieve a state of remission as quickly as possible.

## Author Contributions

TH wrote and MI reviewed the manuscript. All authors contributed to the article and approved the submitted version.

## Conflict of Interest

The authors declare that the research was conducted in the absence of any commercial or financial relationships that could be construed as a potential conflict of interest.

## Publisher’s Note

All claims expressed in this article are solely those of the authors and do not necessarily represent those of their affiliated organizations, or those of the publisher, the editors and the reviewers. Any product that may be evaluated in this article, or claim that may be made by its manufacturer, is not guaranteed or endorsed by the publisher.
